# Correlation between attention deficit/hyperactivity disorder and chronic pain: a survey of adults in Japan

**DOI:** 10.1038/s41598-025-95864-4

**Published:** 2025-04-16

**Authors:** Satoshi Kasahara, Takahiko Yoshimoto, Hiroyuki Oka, Naoko Sato, Taito Morita, Shin-Ichi Niwa, Kanji Uchida, Ko Matsudaira

**Affiliations:** 1 Department of Anesthesiology and Pain Relief Center, The University of Tokyo Hospital, 7-3-1 Hongo, Bunkyo-ku, Tokyo, 113-8655 Japan; 2 Department of Pain Medicine, Fukushima Medical University School of Medicine, Fukushima, Japan; 3 Department of Hygiene, Public Health and Preventive Medicine, Showa University School of Medicine, Tokyo, Japan; 4 Division of Musculoskeletal AI System Development, Faculty of Medicine, The University of Tokyo, Tokyo, Japan; 5 Nursing Department, The University of Tokyo Hospital, Tokyo, Japan; 6 Department of Psychiatry, Aizu Medical Center, Fukushima Medical University, Aizuwakamatsu, Japan; 7 Tailor Made Backpain Clinic, Tokyo, Japan

**Keywords:** Attention deficit/hyperactivity disorder, Autism spectrum disorder, Chronic pain, Epidemiological internet survey, Extreme pain, Pathway analyses, Health care, Risk factors

## Abstract

This cross-sectional epidemiological internet survey assessed whether attention deficit/hyperactivity disorder (ADHD) and autism spectrum disorder (ASD) symptoms are associated with pain chronicity and intensity and explored the relationship between developmental disorder symptoms and pain. Participants were 4028 adults aged 20–64 years who experienced pain, assessed using an 11-point numerical rating scale (NRS), in any body part in the previous 4 weeks. ADHD and ASD symptoms were assessed using the Adult ADHD Self-Report Scale (ASRS) and autism spectrum quotient, respectively. Problems with mental health (PMH) were assessed using the shortened Profile of Mood States. Pathway analyses were performed to examine the association between ADHD symptoms and pain. The chronic pain symptoms (CP) group (*N* = 1465) scored higher than the non-CP group (*N* = 2563) for all ASRS variables. ASRS positivity was associated with CP symptoms and increased with increasing NRS score; the CP group showed particularly high positivity (38.3%) with extreme pain. ADHD symptoms were more strongly associated with CP symptoms and intensity than was PMH (0.26 vs. 0.09). ADHD symptoms, but not ASD symptoms, were associated with CP symptoms. ADHD medications reportedly improve coexisting CP; therefore, ADHD screening and treatment may be important for patients with CP, especially those with extreme pain.

**Supplementary Information:**

The online version contains supplementary material available at 10.1038/s41598-025-95864-4.

## Introduction

In 2022, chronic pain (CP)—based on the International Association for the Study of Pain definition—was first coded as a disease in the International Classification of Diseases (11th edition) (ICD-11)^1,2^. CP was defined as “pain that persists or recurs for > 3 months,” while pain lasting < 3 months was classified as acute pain. A key feature of the ICD-11 CP classification is the introduction of two subcategories: chronic primary pain and chronic secondary pain. Chronic primary pain includes conditions such as fibromyalgia and nonspecific chronic low back pain, where the pain cannot be fully explained by an underlying pathological condition alone. Chronic primary pain refers to pain that persists for > 3 months with significant emotional distress, such as anxiety, depression, or anger, and impairment of daily and social functioning, without apparent tissue damage causing pain^3^. In contrast, chronic secondary pain is attributed to an identifiable organic cause. This classification is aimed at standardizing the diagnosis of CP, which used to be categorized under mental disorders in earlier versions of the ICD, and to clarify treatment strategies, promote research advancements, and ultimately improve patient care. CP can also be influenced by psychosocial factors and has a low improvement rate with the use of usual analgesics, making it an important and challenging issue for society and clinicians^4^.

Recently, studies have focused on the association between attention deficit/hyperactivity disorder (ADHD) and CP classified as chronic primary pain in the ICD-11^5^, such as migraine^6^, fibromyalgia^6,7^, irritable bowel syndrome^6^, chronic low back pain^8–11^, and idiopathic orofacial pain^12–14^. ADHD is a neurodevelopmental disorder characterized by inattention and/or hyperactivity-impulsivity^15^, which is assumed to be due to dopaminergic and noradrenergic nervous system dysfunction^16^. ADHD symptoms are present from childhood, before 12 years of age, and 40–70% of these symptoms persist to various degrees into adulthood^17^. As dopamine and noradrenaline are important for pain perception and control^18^, individuals with ADHD may be more vulnerable to pain^19^. Basic research has also revealed that in rodent models of ADHD, brain abnormalities associated with ADHD lead to central sensitization and hyperalgesia through several neurobiological mechanisms^19–23^. These include increased spontaneous activity in the anterior cingulate cortex–posterior insular cortex pathway due to neuroinflammation, alterations in action potential firing and transmission pathways in the spinal dorsal horn, as well as upregulated norepinephrine synthesis and reduced transmission efficiency. These changes are thought to amplify nociceptive input from the periphery, which ultimately contributes to heightened pain sensitivity.

Studies on pain and ADHD in humans have mostly been conducted in clinical settings. To the best of our knowledge, a study by Stickley et al. is the only epidemiological study conducted in a more general, non-clinical setting. They showed that ADHD symptoms are associated with pain in a general population of 7403 individuals in England^24^. In this study, pain intensity was assessed by asking participants, “During the past 4 weeks, how much did pain interfere with your normal work?” The response options were ‘not at all,’ ‘a little bit,’ ‘moderately,’ ‘quite a bit,’ and ‘extremely.’ For the analysis, the responses were treated as a binary variable, with responses of “extremely” categorized as extreme pain and all other responses categorized as non-extreme pain. Logistic regression analysis revealed ADHD as a significant predictor of extreme pain. However, after adjusting for common mental disorders (CMDs), the association with CMDs was stronger, reducing the effect of ADHD. The authors suggest that CMDs can mediate this relationship, highlighting the importance of CMD screening and treatment in ADHD patients with pain.

However, this study had three key limitations. First, individuals were categorized as having “ADHD symptoms” on the basis of a cutoff score of 14 or higher out of 24 on the Adult ADHD Self-Report Scale (ASRS)^25^, rather than on the basis of the screener score that is specifically designed for ADHD screening. The ASRS screener score (ranging from 0 to 6) has established sensitivity and specificity based on the probability of a clinical ADHD diagnosis at each score level. By utilizing this screener score, it would be possible to estimate the proportion of participants who might meet the criteria for an ADHD diagnosis. Hence, the findings of this study do not allow for an examination of the association between pain and the likelihood of an ADHD diagnosis. Second, the researchers assessed pain by asking about its interference with work, rather than evaluating actual pain occurrence using a validated pain scale. In addition, the authors highlighted the need for more data on pain duration, chronicity, and location, suggesting that future studies collecting such details would provide a clearer understanding of the relationship between pain and ADHD. Third, the need for additional research to determine whether a causal relationship exists between ADHD and pain was emphasized.

Furthermore, ADHD frequently coexists with autism spectrum disorder (ASD)^26^. ASD is characterized by persistent deficits in social interaction and communication, in addition to limited and repetitive patterns of behavior and interest^15^; ASD also has symptoms of sensory hypersensitivity and is reportedly associated with CP^27^. However, in no epidemiological studies have the authors reported on the association between pain and adult developmental disorders, including ADHD and ASD. Furthermore, a potential causal link between ADHD and CP has not been thoroughly examined.

Therefore, we raised two clinical questions: (1) Are ADHD and ASD associated with pain chronicity and intensity, and if so, which has the stronger influence? (2) If developmental disorders and pain are associated, what potential pathways can be explored to understand their relationship? By addressing these questions and bridging the gaps in the literature, we aimed to clarify how symptoms of developmental disorders influence CP. With this objective in mind, we conducted a cross-sectional epidemiological internet survey using large population-based data.

## Results

### Sample characteristics

**Fig. 1:**
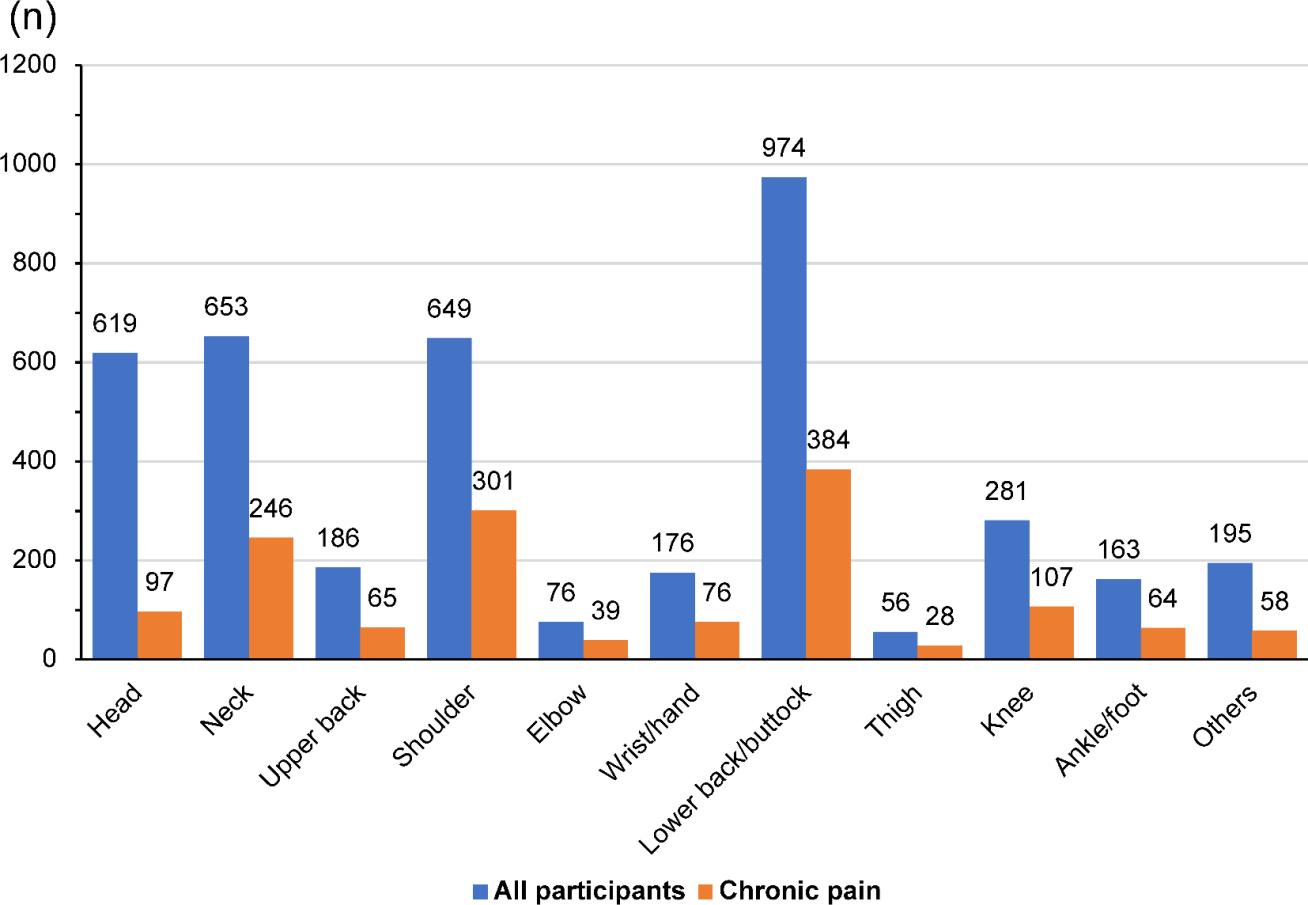
Distribution of participants’ pain areas and chronicity. The blue bars indicate the distribution of the most troublesome body parts among the areas of pain in the last 4 weeks. Among the body sites, the areas in which pain persisted for more than 3 months and became chronic are indicated by orange bars.

Data from all 4028 participants in this study were analyzed; their characteristics are presented in Table 1. Overall, 50.3% of the participants were male, and the median age of all participants was 45 (34–54) years. There were 1465 (36.4%) participants in the CP symptoms group and 2563 (63.6%) in the non-CP symptoms group. The pain intensity in the CP symptoms group was significantly higher [6 (5,7)] than that in the non-CP symptoms group [4 (3,6)]. More participants were married, divorced, or widowed in the CP symptoms group than in the non-CP symptoms group. Problems with physical health (PPH) and problems with mental health (PMH) were significantly more common in the CP symptoms group than in the non-CP symptoms group. The distributions of the most troublesome areas of pain in the last 4 weeks and that of the areas where the pain became chronic are shown in Fig. 1. The top 3 sites for both the most troubling pain areas in the last 4 weeks and the sites where pain became chronic included the back, neck, and shoulders.

**Table 1:** Characteristics of the study participants.

Characteristics	Category		All (*N* = 4028)	Pain symptoms		*P*-value	Significance
				Chronic (*N* = 1465)	Non-chronic (*N* = 2563)		
Sex, n (%)	Male		2026 (50.3)	767 (52.4)	1259 (49.1)	0.05	
	Female		2002 (49.7)	698 (47.6)	1304 (50.9)		
Age, years			45 (34, 54)	48 (38, 56)	42 (32, 52)	< 0.001	***
BMI, kg/m^2^			22.4 (19.5, 24.4)	22.0 (19.7, 24.9)	21.5 (19.5, 24.1)	< 0.001	***
Marital status, n (%)	Married	Count	2175 (54.0)	823 (56.2)	1352 (52.8)	< 0.001	***
		Residual		2.1*	− 2.1*		
	Unmarried	Count	1609 (39.9)	533 (36.4)	1076 (42.0)		
		Residual		− 3.5**	3.5**		
	Divorced or widowed	Count	244 (6.1)	109 (7.4)	135 (5.3)		
		Residual		2.8**	− 2.8**		
Education level, n (%)	No college		1441	938 (64.0)	1649 (64.3)	0.843	
	College		2587	527 (36.0)	914 (35.7)		
Employment type, n (%)	Regular		1203	419 (28.6)	784 (30.6)	0.413	
	Non-regular		796	295 (20.1)	501 (19.5)		
	Non-worker		2029	751 (51.3)	1278 (49.9)		
Problems with physical health, n (%)	Yes		1510	625 (42.7)	885 (34.5)	< 0.001	***
	No		2518	840 (57.3)	1678 (65.5)		
Problems with mental health, n (%)	Yes		543	242 (16.5)	301 (11.7)	< 0.001	***
	No		3485	1223 (83.5)	2262 (88.3)		
Pain NRS			5 (3, 6)	6 (5, 7)	4 (3, 6)	< 0.001	***

Variables are shown as medians (25th and 75th percentiles), except where indicated as n (%). *P < 0.05, **P < 0.01, ***P < 0.001. BMI, body mass index; NRS, numerical rating scale.

### Association between ADHD and ASD symptoms and pain

**Fig. 2:**
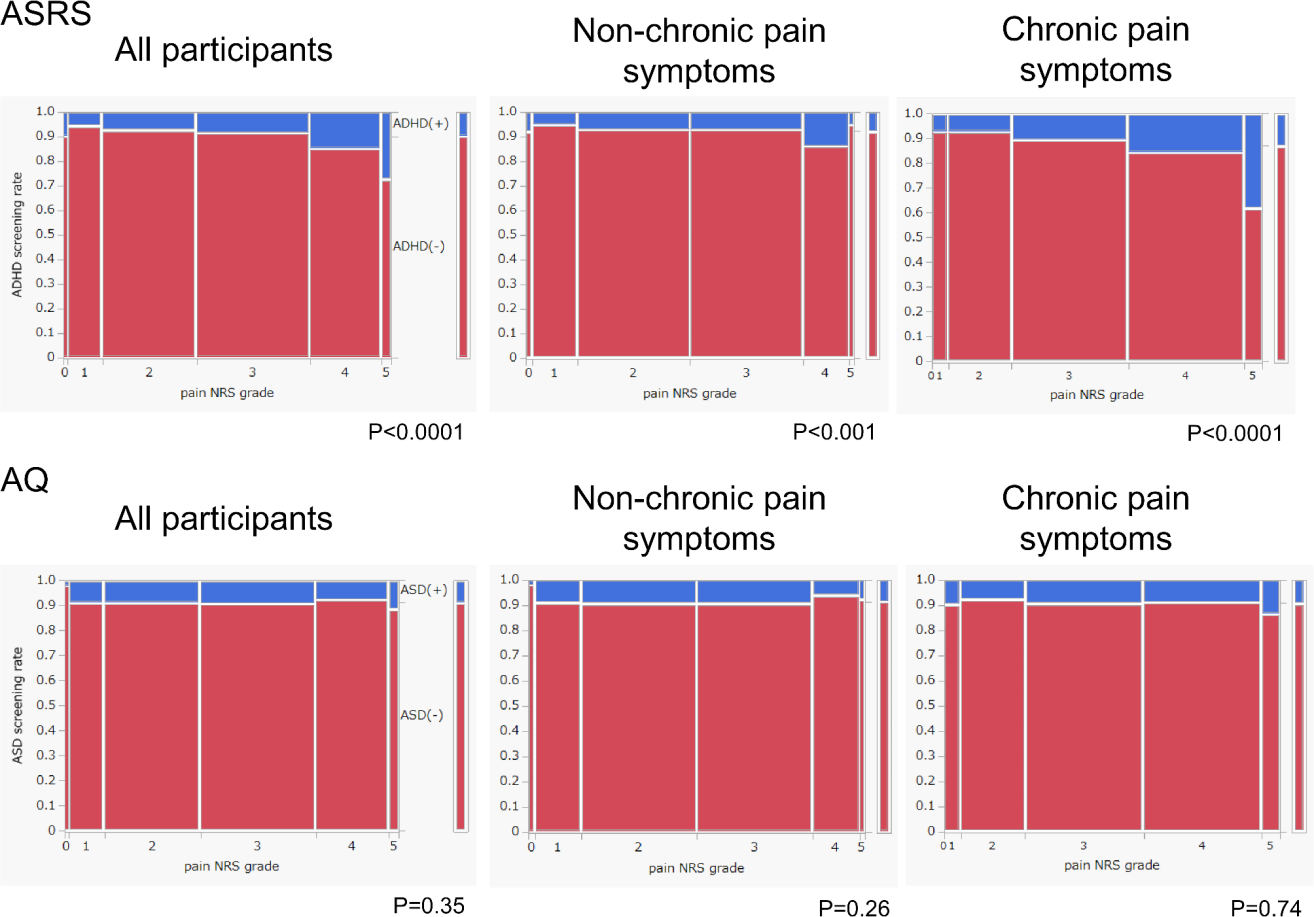
Association between the pain NRS scores and ASRS or AQ screening positivity rates in the entire cohort, non-CP symptoms group, and CP symptoms group. The width of each pain NRS grade on the horizontal axis represents the proportion of individuals who reported that grade of pain. The ASRS screening positivity rate increases significantly with increasing pain NRS score, especially for grade 5 in the CP group, with a positivity rate of 38.3%. ADHD, attention deficit/hyperactivity disorder; AQ, autism spectrum quotient; ASD, autism spectrum disorder; ASRS, adult ADHD self-report scale; NRS, numerical rating scale; CP, chronic pain.

The CP symptoms group had significantly higher ASRS scores than the non-CP symptoms group did for all variables, although no significant differences were noted in autism spectrum quotient (AQ) scores between the groups (Table 2). Significantly fewer participants with CP symptoms were categorized as ADHD and ASD symptom-negative, and significantly more participants with CP symptoms were categorized as only ADHD symptom-positive, according to the adjusted residuals; that is, screening positive for only ADHD symptoms was significantly associated with CP symptoms. Regarding the ASRS score, ADHD symptom positivity increased significantly with increasing pain stage in the entire cohort, non-CP symptoms group, and CP symptoms group (Fig. 2). In the CP symptoms group, 38.3% of respondents with pain reported as numerical rating scale (NRS) grade 5 (NRS score = 9–10) screened positive for ADHD symptoms. Conversely, no significant association was noted between pain grade and screening positive for ASD symptoms in the entire cohort, non-CP symptoms group, and CP symptoms group.

**Table 2:** Detailed results of the developmental disabilities scale.

Rating scale	Category		All (*N* = 4028)	Pain symptoms		*P*-value	Bonferroni correction (*P*)	Significance
				Chronic (*N* = 1465)	Non-chronic (*N* = 2563)			
ASRS	Screening positive, n (%)		398 (9.9)	191 (13.0)	207 (8.1)	< 0.001	< 0.001	***
	Q6 total (0–24)		7 (5, 10)	8 (5, 11)	7 (4, 10)	< 0.001	< 0.001	***
	Q18 total (0–72)		19 (12, 27)	20 (13, 29)	19 (11, 26)	< 0.001	< 0.001	***
	Inattention (0–36)		11 (7, 15)	12 (8, 16)	11 (7, 15)	< 0.001	< 0.001	***
	Hyperactivity-impulsivity (0–36)		8 (4, 12)	9 (4, 13)	8 (4, 11)	< 0.001	< 0.001	***
AQ	Screening positive, n (%)		356 (8.8)	136 (9.3)	220 (8.6)	0.45	1.00	
	Q50 total (0–50)		24 (19, 28)	24 (18, 28)	24 (19, 28)	0.40	1.00	
	Communication (0–10)		4 (2, 6)	4 (2, 6)	4 (2, 6)	0.50	1.00	
	Social skills (0–10)		6 (4, 8)	6 (4, 8)	6 (4, 8)	0.05	0.80	
	Imagination (0–10)		4 (3, 6)	4 (3, 6)	4 (3, 6)	0.29	1.00	
	Attention to detail (0–10)		4 (3, 6)	4 (3, 6)	4 (3, 6)	0.81	1.00	
	Attention switching (0–10)		5 (4, 6)	5 (3, 6)	5 (4, 6)	0.54	1.00	
ADHD/ASD symptom screening	Both ADHD and ASD symptom-negative	Count	3366	1181 (35.1%)	2185 (64.9%)	< 0.001	< 0.01	**
		Residual		− 3.82	3.82			
	Only ADHD symptom-positive	Count	306	148 (48.4%)	158 (51.6%)	< 0.001	< 0.001	***
		Residual		4.54	− 4.54			
	Only ASD symptom-positive	Count	264	93 (35.2%)	171 (64.8%)	0.69	1.00	
		Residual		− 0.40	0.40			
	Both ADHD and ASD symptom-positive	Count	92	43 (46.7%)	49 (53.3%)	<0.05	0.60	
		Residual		2.08	− 2.08			
			4028	1465 (36.4%)	2563 (63.6%)			

Variables are shown as medians (25th and 75th percentiles), except where indicated as n (%). **P < 0.01, ***P < 0.001. ADHD, attention deficit/hyperactivity disorder; AQ, autism spectrum quotient; ASD, autism spectrum disorder; ASRS, adult ADHD self-report scale.

Multivariate logistic regression analysis using the binary pain variable (having extreme pain or not) as the outcome revealed a significant association between ADHD symptom positivity and extreme pain (Table 3). In an unadjusted model where the covariate was ADHD symptoms only (Model 1), the odds of having extreme pain were over threefold higher for those who were ADHD symptom-positive than for those who were ADHD symptom-negative (OR: 3.68, 95% confidence interval [CI] 2.43–5.58). In the unadjusted model with ASD symptoms as the only covariate (Model 2), there was no association between extreme pain and ASD symptoms (OR: 1.38, 95% CI 0.78–2.43). In the model with ADHD and ASD symptoms as covariates (Model 3), the odds of having extreme pain were significant only for ADHD symptom-positive participants (OR: 3.68, 95% CI 2.40–5.63). Even after adjusting for all the demographic variables (Model 4) and including PPH (Model 5), the odds of ADHD symptoms being associated with extreme pain remained at or above 3.0. However, when PMH was included in the analysis, the odds of ADHD symptom-positive individuals reporting extreme pain was significantly attenuated (Models 6 and 7). In the fully adjusted model (Model 7), the OR decreased to 2.73 (1.73–4.31). Hence, ADHD symptoms were significantly associated with having extreme pain in all models that included it as a variable (Models 1, 2–7), although the association was significantly attenuated when PMH was included (Models 6 and 7).

**Table 3:** Association between ADHD and extreme pain, as estimated using binary logistic regression.

Characteristics	Model 1	Model 2	Model 3	Model 4	Model 5	Model 6	Model 7
ADHD symptoms^a^	3.68***		3.68***	3.49***	3.44***	2.66***	2.73***
	[2.43, 5.58]		[2.40, 5.63]	[2.27, 5.37]	[2.24, 5.30]	[1.70, 4.17]	[1.73, 4.31]
ASD symptoms^a^		1.38	1.00				0.81
		[0.78, 2.43]	[0.56, 1.81]				[0.44, 1.51]
Age				0.98	0.98	0.98	0.98
				[0.96, 1.00]	[0.96, 1.00]	[0.96, 1.00]	[0.96, 1.00]
Sex							
Male				ref.	ref.	ref.	ref.
Female				1.26	1.27	1.25	1.25
				[0.81, 1.96]	[0.82, 1.98]	[0.80, 1.95]	[0.80, 1.95]
BMI				1.74**		1.66*	1.64*
				[1.14, 2.65]		[1.09, 2.54]	[1.06, 2.54]
Marital status							
Unmarried				ref.	ref.	ref.	ref.
Married				1.65*	1.65*	1.80*	1.77*
				[1.05, 2.61]	[1.05, 2.61]	[1.13, 2.87]	[1.11, 2.82]
Divorced or widowed				1.57	1.55	1.64	1.62
				[0.69, 3.58]	[0.68, 3.53]	[0.72, 3.77]	[0.70, 3.71]
Educational background^b^							
College				ref.	ref.	ref.	ref.
No college				1.51*	1.51*	1.49*	1.49*
				[1.03, 2.21]	[1.03, 2.21]	[1.02, 2.19]	[1.02, 2.19]
Employment type^c^							
Regular				ref.	ref.	ref.	ref.
Non-regular				1.32	1.32	1.31	1.33
				[0.75, 2.31]	[0.76, 2.32]	[0.75, 2.31]	[0.76, 2.34]
Non-worker				1.10	1.11	1.09	1.11
				[0.66, 1.83]	[0.67, 1.85]	[0.66, 1.83]	[0.67, 1.86]
Problems with physical health^d^					1.16		1.06
					[0.78, 1.73]		[0.71, 1.59]
Problems with mental health^e^						2.85***	2.90***
						[1.88, 4.32]	[1.90, 4.42]

Data are presented as odds ratios (ORs) and 95% confidence intervals (CIs). *P < 0.05, **P < 0.01, ***P < 0.001.

Each model was adjusted for all covariates in each column. Seven models were constructed: Model 1: univariate analysis of ADHD symptoms (unadjusted); Model 2: univariate analysis of ASD symptoms (unadjusted); Model 3: adjusted for ASD symptoms; Model 4: adjusted for age, sex, BMI, marital status, educational background, and employment status; Model 5: adjusted for the covariates in Model 4 and problems with physical health; Model 6: adjusted for the covariates in Model 4 and problems with mental health; Model 7: adjusted for the covariates in Model 4 and ASD symptoms, problems with physical health, and problems with mental health (fully adjusted model).

Responses with an average NRS score of 9–10 for the pain area that was most troubling within 4 weeks were considered “extreme pain,” and other responses were considered “no extreme pain” and were the dependent variables in the binary logistic regression analysis.

^a^The presence of ADHD and ASD symptoms corresponded to positive and negative ASRS and AQ screening, respectively.

^b^Responses of graduation from junior high school, high school, or vocational high school were considered “non-college,” while those of graduation from vocational school, university, or postgraduate school were considered “college.”

^c^Those who answered that they were regular employees were classified as “regular”; those who answered that they were part-timers, temporary workers, running a business, helping out in the family business, or working at home as an in-house worker or a freelancer were classified as “non-regular”; and those who answered that they were students, housewives/househusbands, unemployed (including retired and job-seeking), and others (none of the above) were considered “non-workers.”

^d^A person was considered to have a physical health problem if they had been diagnosed by a doctor in the past with 1–12 diseases (hypertension, hyperlipidemia, diabetes, obesity, rheumatoid arthritis, liver dysfunction, renal dysfunction, atopic dermatitis, bronchial asthma, postherpetic neuralgia, malignancy, and others).

^e^Participants who scored 75 (clinical psychiatric level) or higher on any one of the five subscales of the POMS (tension-anxiety (T–A), depression–dejection (D), anger–hostility (A–H), fatigue (F), and confusion (C)) were considered to have mental health problems.

ADHD, attention deficit/hyperactivity disorder; AQ, autism spectrum quotient; ASD, autism spectrum disorder; ASRS, Adult ADHD Self-Report Scale; BMI, body mass index; POMS, Profile of Mood States; ref., reference category; NRS, numerical rating scale.

### Association between ADHD symptoms and CP symptoms and intensity

**Fig. 3:**
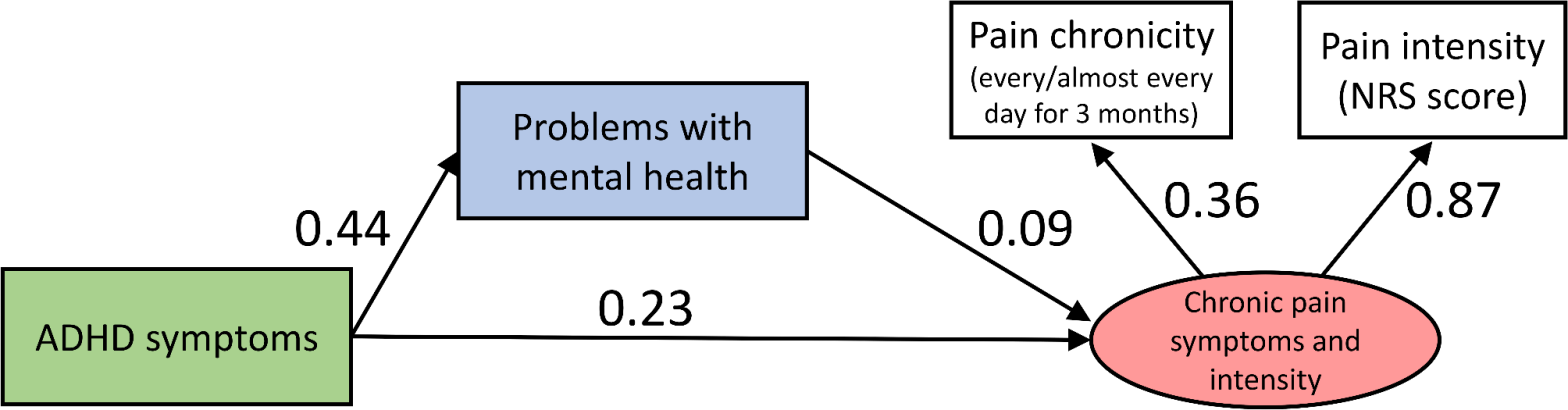
Pathway diagram showing the relationship between ADHD symptoms and PMH and CP symptoms and intensity. The potential variable, CP symptoms and intensity, was defined as pain chronicity and pain intensity. In the pathway, the starting factor affects the factors indicated by the arrows. Error variables are not shown. Pathway coefficients are shown as standardized coefficients; all coefficients are significant at *P* < 0.001. The goodness-of-fit of the model was impeccable, with χ^2^ = 0.135, df = 1, *P* = 0.244, GFI = 1.000, CFI = 1.000, TLI = 0.999, and RMSEA = 0.009 for all indicators. The diagram shows the direct effect of ADHD symptoms on CP symptoms and intensity, with a pathway coefficient of 0.23. The pathway coefficients for the direct effect of ADHD symptoms on PMH and of PMH on CP symptoms and intensity were 0.44 and 0.09, respectively. The pathway coefficient for the indirect effect of ADHD symptoms on CP symptoms and intensity was 0.04, and the pathway coefficient for the overall effect of ADHD symptoms on CP symptoms and intensity, including the direct and indirect effects, was 0.26. ADHD, attention deficit/hyperactivity disorder; NRS, numerical rating scale; PMH, problems with mental health; CP, chronic pain; GFI, goodness-of-fit index; CFI, comparable fit index; RMSEA, root mean square error of approximation; TLI, Tucker–Lewis Index.

ADHD symptoms were associated with both PMH and CP symptoms and intensity. The overall association between ADHD symptoms and CP symptoms and intensity (0.26; indirect effect 0.04 + direct effect 0.23) was stronger than the association between PMH and pain (0.09) (Fig. 3; Tables 4 and 5).

**Table 4:** Path analysis results.

Path		β	B	SE	*P*-value
Direct effect	ADHD symptoms → Chronic pain symptoms and intensity	0.225	0.003	0.001	< 0.001
	ADHD symptoms → Problems with mental health	0.439	0.823	0.027	< 0.001
	Problems with mental health → Chronic pain symptoms and intensity	0.090	0.001	< 0.001	< 0.001
Indirect effect	ADHD symptoms → Problems with mental health → Chronic pain symptoms and intensity	0.039	0.001		

ADHD, attention deficit/hyperactivity disorder; β, standardized path coefficient; B, unstandardized path coefficient; SE, standard error.

**Table 5:** Model fit indices.

Fit index	Value	Recommended criteria
χ^2^ (df)	1.35 (1), *p* = 0.244	*p* > 0.05
GFI	1.000	≥ 0.90
CFI	1.000	≥ 0.90
TLI	0.999	≥ 0.90
RMSEA (90% CI)	0.009 (0.001, 0.044)	≤ 0.08

χ^2^, chi-square values; df, degree of freedom; CI, confidence interval; CFI, comparative fit index; GFI, goodness-of-fit index; RMSEA, root mean square error of approximation; TLI, Tucker–Lewis Index.

## Discussion

Herein, we found that ADHD symptoms were associated with pain chronicity and intensity, and we formulated a statistical model to examine the potential relationship between ADHD symptoms and CP symptoms and intensity.

First, the screening positivity rates for both the AQ and ASRS among participants with pain (ASD symptoms, 8.8%; ADHD symptoms, 9.9%) were higher than those in the general population found in a previous internet study (ASD symptoms, 4.3%; ADHD symptoms, 7.2%)^28^. Moreover, contrary to ASD symptoms, ADHD symptoms were associated with pain chronicity and intensity (especially extreme pain). As noted previously^27^, people with ASD symptoms may be less sensitive to pain.

Patients with extreme pain are known to have a longer history of pain, longer functional disability, more surgical procedures and hospitalizations, higher rates of drug dependence, more pronounced pain behaviors, and less responsiveness to treatment than do those with mild pain^29^. Recent epidemiological studies on the economic cost of CP have revealed that the direct cost of healthcare utilization is 1.78–4.45 times higher, the indirect cost of lost productivity 34.62 times higher, and the total cost 15.96 times higher for people with extreme pain than for those with mild pain^30,31^. The excess annual cost would be €308.88 for mild pain and €4928.70 for extreme pain per case, making this an important social issue^31^. The finding of an association between extreme pain and ADHD symptoms is important from both medical and economic perspectives and useful for the future development of new approaches.

Furthermore, herein, the association between ADHD symptoms and pain was mediated by PMH, which is consistent with the findings of a previous study^24^. Adults with ADHD are more likely to have comorbid psychiatric disorders, including depression and anxiety disorders and PMH^32^, which could exacerbate pain.

Second, a significant statistical model was identified, which suggested a potential association between ADHD symptoms and pain, with ADHD symptoms accounting for approximately 26% of the variance in CP symptoms and intensity. Traditionally, it has been assumed that CP is caused by the presence of PMH, such as anxiety or depression^33^, as ADHD has not been included in the discussion of the association between CP and mental illness. However, the consideration of ADHD symptoms in the present study revealed that its association with CP symptoms and intensity (0.26) was stronger than that of PMH (0.09). This finding is consistent with our multivariate logistic regression analysis results, which showed a significant association of ADHD symptoms with extreme pain after adjusting for PMH, although it was attenuated by PMH. The authors of previous studies have stated that screening and intervention for CMDs are important because such disorders mediate the association between pain and ADHD symptoms^24^. Our results suggest that screening and intervention for ADHD symptoms should also be considered.

The ASRS scores were significantly higher in the CP symptoms group than in the non-CP symptoms group, particularly among those who reported extreme pain, with a positive ADHD symptom screening rate of 38.3%. The ASRS has a sensitivity and specificity of 68.7% and 99.5%, respectively, indicating relatively low sensitivity and a high false-negative rate^25^. If the participants’ ADHD symptoms had been clinically assessed, an estimated 57.45% of those with extreme pain in the CP symptoms group might have met the criteria for a theoretical diagnosis of adult ADHD. Although the theoretical ADHD diagnostic rate of 57.45% among those with extreme pain may appear high, it could be considered plausible given that the comorbidity of ADHD in individuals with refractory chronic primary pain in clinical settings has been reported to be 72.5%^5^. CP and ADHD are similar in terms of dysfunction of the dopaminergic and noradrenergic nervous systems^16,18^. ADHD medications activate the dopaminergic and noradrenergic nerves in the brain^34^, and recent human studies have revealed that ADHD medications improve CP comorbid with ADHD^5,10–14,35^. Additionally, it has been shown that ADHD medications improve the increased blood flow in the precuneus of individuals with CP comorbid with ADHD, which may consequently lead to the improvement of reduced blood flow in the prefrontal cortex^36^. It has also been demonstrated in rodent models of ADHD that hyperalgesia due to central sensitization, along with ADHD symptoms such as hyperactivity and inattention, can be improved by atomoxetine, a selective norepinephrine reuptake inhibitor used for ADHD treatment^20,23^. Currently, however, 80% of adult ADHD cases are missed, even in psychiatric practice^37^. Therefore, proactive ADHD screening and treatment could be beneficial for patients with CP, especially those who experience extreme pain; this may also contribute to reducing economic costs.

Furthermore, the association between ADHD and pain can be considered from an evolutionary and genetic perspective. The seven-repeat subclass (DRD4-7R) of the dopamine receptor *DRD4*, one of the genes responsible for ADHD, is associated with novelty-seeking and is known to correlate with the distance of human migration from Africa^38^. Perhaps because ADHD is characterized by risk-taking and venturing into uncharted territory, patients with the condition may be acutely sensitive to pain, a signal indicating potential injury, illness, or physical discomfort.

This study has four limitations. The first is the potential sampling bias in participant recruitment through web-based surveys, such as in this study, wherein individuals without internet access cannot participate. Registrants interested in their health may have been more likely to respond to our survey. Therefore, participants may have overestimated their pain and ADHD symptoms, leading to exaggerated effects of ADHD symptoms on CP symptoms. Furthermore, it should be noted that the registered participants were limited to those who reported pain in the 4 weeks before the time of the survey, and individuals who did not report pain were not included. Second, ADHD symptoms in this study may have been non-clinical, as they were self-reported rather than based on a clinical diagnosis. Moreover, the ADHD and ASD symptoms assessed in this study using standardized scales reflect current symptoms only and do not confirm their presence since childhood. The diagnosis of developmental disorders requires verification that symptoms have been present since early childhood. Therefore, the ADHD and ASD symptoms identified in this study cannot be equated with a clinical diagnosis of ADHD or ASD. Third, the inattention and hyperactivity/impulsivity classified as ADHD symptoms in this study, as well as the communication difficulties and restricted/repetitive behaviors classified as ASD symptoms, are not exclusively observed in individuals with ADHD or ASD. These symptoms could result from other conditions, such as mood disorders, anxiety disorders, schizophrenia spectrum disorders, intellectual disabilities, or language disorders, which could contribute to difficulties in social situations^39^. Fourth, as this study had a cross-sectional design, the temporal order of factors in the path analysis remains unclear, and unmeasured confounding factors cannot be ruled out. Additionally, bidirectional causal relationships between factors are conceivable. Therefore, definitive conclusions about causality cannot be drawn solely based on the findings of this study. These points should be considered when generalizing our findings.

In conclusion, although previous studies on the association between ADHD, ASD, and CP are limited, this study provides a detailed analysis of the relationship between ADHD symptoms and CP symptoms, demonstrating that ADHD symptoms are particularly associated with pain severity. In contrast, ASD symptoms did not show a clear association with CP symptoms. This study was conducted using a large-scale internet survey and a sample of 4028 adults, incorporating a relatively diverse population. This aspect suggests that the findings of this study are not restricted to a specific group but may be generalizable to a broader population. Furthermore, given previous reports that ADHD treatment may influence CP management, our study provides empirical evidence supporting the potential utility of ADHD screening and treatment considerations in the clinical management of CP and relevant policymaking. Psychiatrists, pain physicians, orthopedic specialists, and professionals who are involved in occupational health, welfare, and policy may benefit from these findings. Screening and intervention for PMH and ADHD could play a role in managing CP. Future research on the relationship between ADHD and CP is required, including clinical longitudinal studies and research on the biological mechanisms of pain from the perspective of ADHD.

## Methods

### Study population

We conducted a cross-sectional web-based epidemiological study among Japanese adults from July 29, 2020, to August 19, 2020. The participants were recruited through internet research agency Hamon Co. (Kanagawa, Japan) using the JAPAN Cloud Panel (https://gmo-research.ai/service/panel/jcp), a large-scale online research panel operated by GMO Research, Inc. This panel consists of approximately 23.94 million registered monitors as of August 2023, covering a diverse range of demographic backgrounds. The sample included individuals aged 20–64 years, with a balanced distribution of sex (male and female) and geographic regions across Japan. The panel members have provided detailed demographic information, including occupation, household income, educational background, and lifestyle habits. Furthermore, periodic updates and screening surveys ensure that the registered data remain current and reliable.

The participants were selected based on predefined inclusion criteria: (1) age 20–64 years and (2) experience of pain in any body part in the previous 4 weeks. The inclusion age is based on the Japanese age standards (adults = 20 years; older adults = 65 years) given that the study population included working adults. Pain in any body part was considered, as CP can occur in various body parts^40^. In studies assessing pain prevalence^41^, the customary timepoints used are “at the time of the survey,” “after 4 weeks,” or “after 1 year”—we employed the most universally used period (4 weeks). The exclusion criteria were (1) inability to provide consent, (2) age < 20 years or ≥ 65 years, and (3) absence of physical pain in the previous 4 weeks. This recruitment method, conducted in collaboration with Hamon Co., ensures a broad representation of the Japanese population while maintaining high data reliability through rigorous quality control measures implemented by the research panel provider.

Recruitment emails were sent to 103,556 panelists who were stratified according to sex and age. Questionnaires were accessible to those who consented to participate. Missing or incomplete responses were automatically rejected, generating no missing data. Participants who completed the questionnaire were awarded reward points worth 0.52 USD as an incentive. The reported prevalence of high-impact CP (HICP) is 4.8%^40^; the sample size was set at 4000 to ensure 200 HICP events, allowing for multivariate analysis. The survey was closed after reaching 4028 respondents, determined broadly according to the Japanese population distribution (sex and age).

This study was approved by the Institutional Review Board of The University of Tokyo (No. 2019296NI), and informed consent for participation in this study was obtained from all the study participants.

### Measurements

#### Pain

Participants were asked about their pain, the areas where they had experienced pain in the last 4 weeks (using manikin drawings, with each of the 11 body parts assigned a color; multiple areas could be selected), and the area of pain that troubled them most. For the most troublesome area, the average pain intensity was assessed using an 11-point NRS^42^ (0 = no pain, 10 = most extreme pain).

#### Pain chronicity

Participants were asked how often over the last 3 months they experienced pain at any site where they had pain in the last 4 weeks. The possible responses were: (1) never, (2) for a few days, (3) almost every day, and (4) every day. Based on the ICD-11 definition of CP mentioned earlier, participants who answered with responses (3) and (4) were included in the CP symptoms group, and those who answered with responses (1) and (2) were included in the non-CP symptoms group. Additionally, participants were asked to indicate, using the 11 color-coded areas on the manikin diagrams, areas where they experienced persistent pain for > 3 months.

#### ADHD symptoms

ADHD symptoms were assessed using the ASRS^25^, which was developed by the World Health Organization and is freely available. The ASRS comprises 9 inattentive and 9 hyperactive-impulsive questions corresponding to the Diagnostic and Statistical Manual of Mental Disorders, Fourth Edition, Text Revision ADHD diagnostic criteria. Each question is scored on a 5-point scale ranging from 0 for “never” to 4 for “very often,” and an inattention score (0–36), hyperactivity-impulsivity score (0–36), and Q18 total score (0–72) are derived. In the screener consisting of 6 (4 inattentive and 2 hyperactive-impulsive) of these questions, a positive result was obtained if four or more answers were within the set score range and a diagnosis of adult ADHD was highly possible. The reported sensitivity and specificity of the ASRS screener are 68.7% and 99.5%, respectively^25^.

#### ASD symptoms

ASD symptoms were assessed using the AQ^43^, which comprises 50 questions and a score range of 0–50. The AQ has five subscales: social skills, attention switching, attention to detail, communication, and imagination. A score of ≥ 33 is considered a positive screening score and a likely diagnosis of ASD. The Japanese version has a reported sensitivity and specificity of 87.8% and 97.4%, respectively^43^.

#### Problems with physical health

Information on the following physician-diagnosed medical problems was collected: hypertension, hyperlipidemia, diabetes, obesity, rheumatoid arthritis, liver dysfunction, renal dysfunction, atopic dermatitis, bronchial asthma, postherpetic neuralgia, malignancy, and others. Participants who selected any of these items were judged to have PPH.

#### Problems with mental health

Mood states were assessed using the short version of the Profile of Mood States (POMS)^44^, which comprises 30 questions and has 6 subscales (Tension-Anxiety, Depression-Dejection, Anger-Hostility, Vigor, Fatigue, and Confusion). Participants with a T-score of ≥ 75 on any of the five negative subscales—Tension-Anxiety, Depression-Dejection, Anger-Hostility, Fatigue, or Confusion—indicating a clinical psychiatric level, were judged to have PMH. Additionally, in the path analysis, we calculated the Total Mood Disturbance (TMD) score, a comprehensive indicator of mood disorders, based on the scores of each POMS subscale^45^. The TMD score was computed as follows: TMD = (Tension-Anxiety + Depression-Dejection + Anger-Hostility + Fatigue + Confusion) – Vigor.

### Statistical analyses

Data normality was assessed using the Kolmogorov–Smirnov–Lilliefors test; no variables were normally distributed. Hence, all continuous variables are presented as medians (25th percentile, 75th percentile). The participant characteristics were compared between the CP symptoms and non-CP symptoms groups using the Wilcoxon rank-sum test for continuous variables and chi-square or Fisher exact tests for categorical variables. Additionally, because ADHD symptoms and ASD symptoms can coexist, a chi-square test was conducted on the association between the combination of ADHD and ASD symptom screening results and CP symptoms. For marital status and combined ADHD and ASD symptom screening results, adjusted residuals were calculated, and significant differences were found.

To examine the association between pain intensity stages and the percentage of positive screening for ADHD and ASD symptoms, the chi-square test was conducted; the NRS score was categorized as follows: 0 for grade 0, 1–2 for grade 1, 3–4 for grade 2, 5–6 for grade 3, 7–8 for grade 4, and 9–10 for grade 5.

To examine the association between pain intensity and ADHD and ASD symptoms, ORs and 95% CIs were calculated using multivariate logistic regression analysis. Previous epidemiological studies have shown that extreme pain has a significant impact on the economic cost of CP^30,31^. In line with previous research^24^, we dichotomized the outcome of the logistic regression analysis by classifying an NRS score of 9–10 as “with extreme pain” and 0–8 as “without extreme pain.” The covariates included variables that were reported as significant in a previous study^24^—age, sex, education, and working economic status—as well as body mass index and marital status, which showed significant differences in terms of CP symptoms in the present study. A hierarchical analysis was conducted wherein each block of variables was entered sequentially into the model to adjust for confounding factors, and seven different models were constructed.

Based on the logistic regression results, a pathway analysis was conducted to examine the causal relationship between pain, ADHD symptoms, and PMH. ADHD symptoms were assessed using the total ASRS Q18 score (0–72 points). PMH was evaluated using the TMD score of the POMS (− 20 to 100 points). Pain chronicity was defined as a binary variable based on whether the pain frequency was “every/almost every day for 3 months” or not. Pain intensity was assessed using the NRS score for pain (0–10 points). For the identification of paths in the model, the best-fitting model was selected based on the fit indices (BCC0, BIC0, C/df, p-value) obtained from the analysis using the “Exploratory Model Specification” command in AMOS 28 (Supplementary File S1). The goodness-of-fit of the overall model was assessed using chi-square values, the goodness-of-fit index, comparative fit index, Tucker–Lewis Index, and root mean square error of approximation.

All statistical analyses were performed using JMP Pro version 16 (SAS Institute Japan, Tokyo, Japan) and AMOS 28 (IBM, Armonk, NY). Statistical significance for all analyses was set at *P* < 0.05 with a two-tailed test. For multiple comparisons, Bonferroni correction was used.

### Transparency declaration

The guarantor, Satoshi Kasahara, affirms that this manuscript is an honest, accurate, and transparent account of the study being reported. No important aspects of the study have been omitted, and any discrepancies from the study as originally planned have been explained.

## Electronic supplementary material

Below is the link to the electronic supplementary material.

Supplementary Material 1.

## Data Availability

The datasets generated and/or analyzed during the current study are available from the corresponding author on reasonable request.
