# Supplementary material for: Correlation between attention deficit/hyperactivity disorder and chronic pain: a survey of adults in Japan
Source: Sci Rep. 2025 Apr 16;15:13165. doi: 10.1038/s41598-025-95864-4 (PMC12003818; doi:10.1038/s41598-025-95864-4)
Supplement: Supplementary file 1 — Supplementary Material 1. [file 41598_2025_95864_MOESM1_ESM.pdf]

Exploratory Model Specification

Based on the results of the logistic regression analysis (Table 3), paths a, b, and c were specified as shown in the figure. Subsequently, an analysis was conducted using the "Exploratory Model Specification" command in AMOS 28.

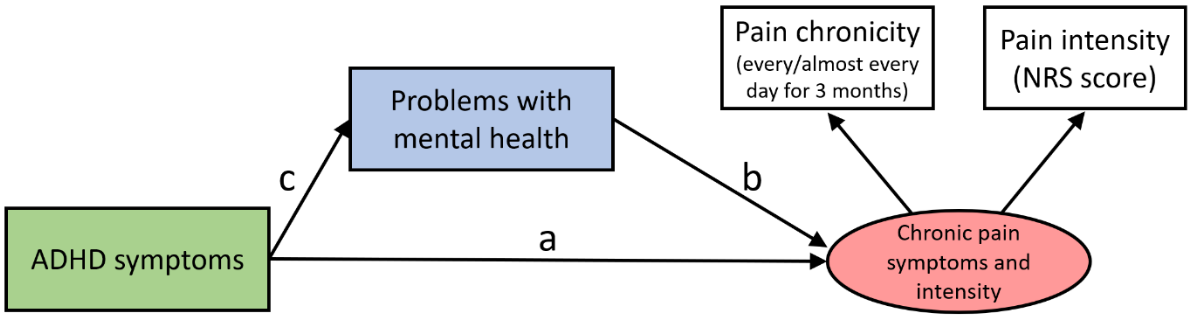

As a result, the fit indices for each model (Models 1 to 8) were output. The best fit for each column of fit indices (BCC0, BIC0, C/df, p-value) is underlined and highlighted in bold font. Ultimately, we selected Model 8 as the final model.

Model	Path			Parameter	df	C	C-df	BCC 0	BIC 0	C/df	p	Annotation
	a	b	c									
1	-	-	-	6	4							Unidentified
2	+	-	-	7	3	884.974	881.974	879.611	867.014	294.991	0.000	
3	-	+	-	7	3	994.799	991.799	989.435	976.838	331.6	0.000	
4	-	-	+	7	3							Unidentified
5	+	-	+	8	2	22.515	20.515	19.154	12.855	11.257	0.000	
6	-	+	+	8	2	132.34	130.34	128.979	122.68	66.17	0.000	
7	+	+	-	8	2	863.818	861.818	860.457	854.158	431.909	0.000	
8	+	+	+	9	1	1.358	0.358	**0.000**	**0.000**	**1.358**	**0.244**	
Saturated model				10	0	0	0	0.644	6.943			
